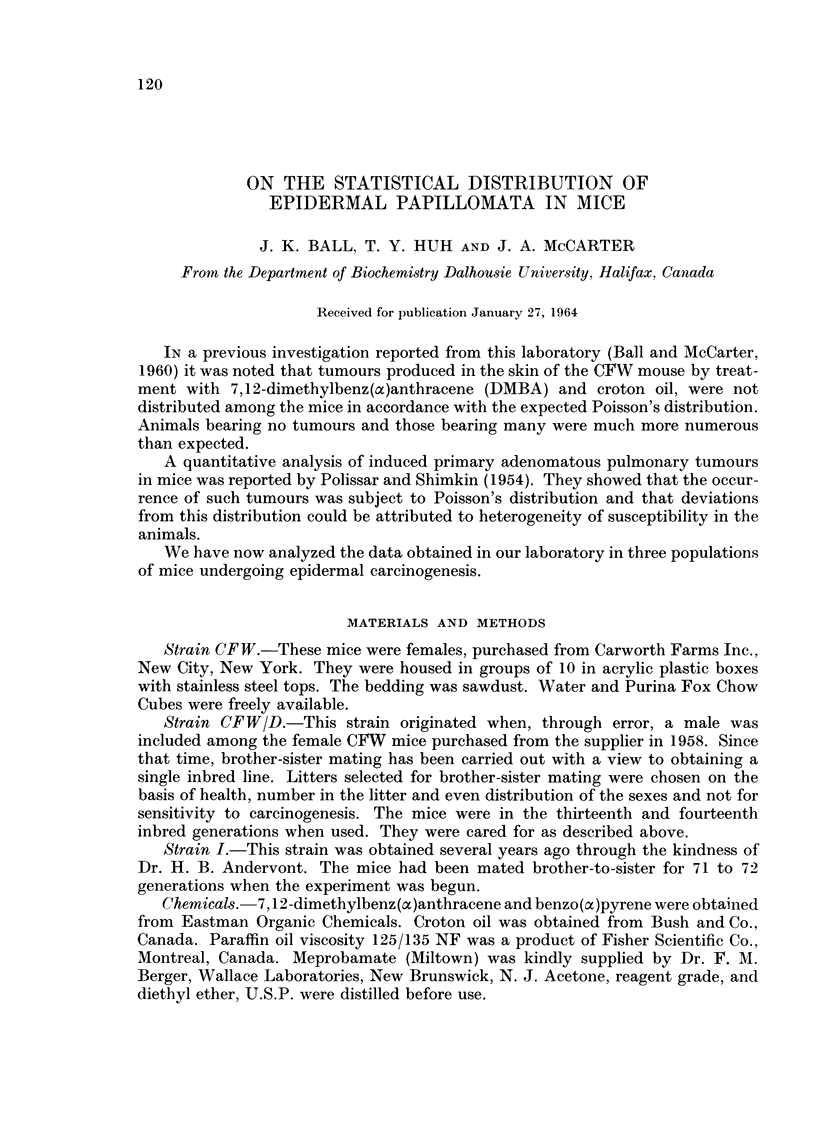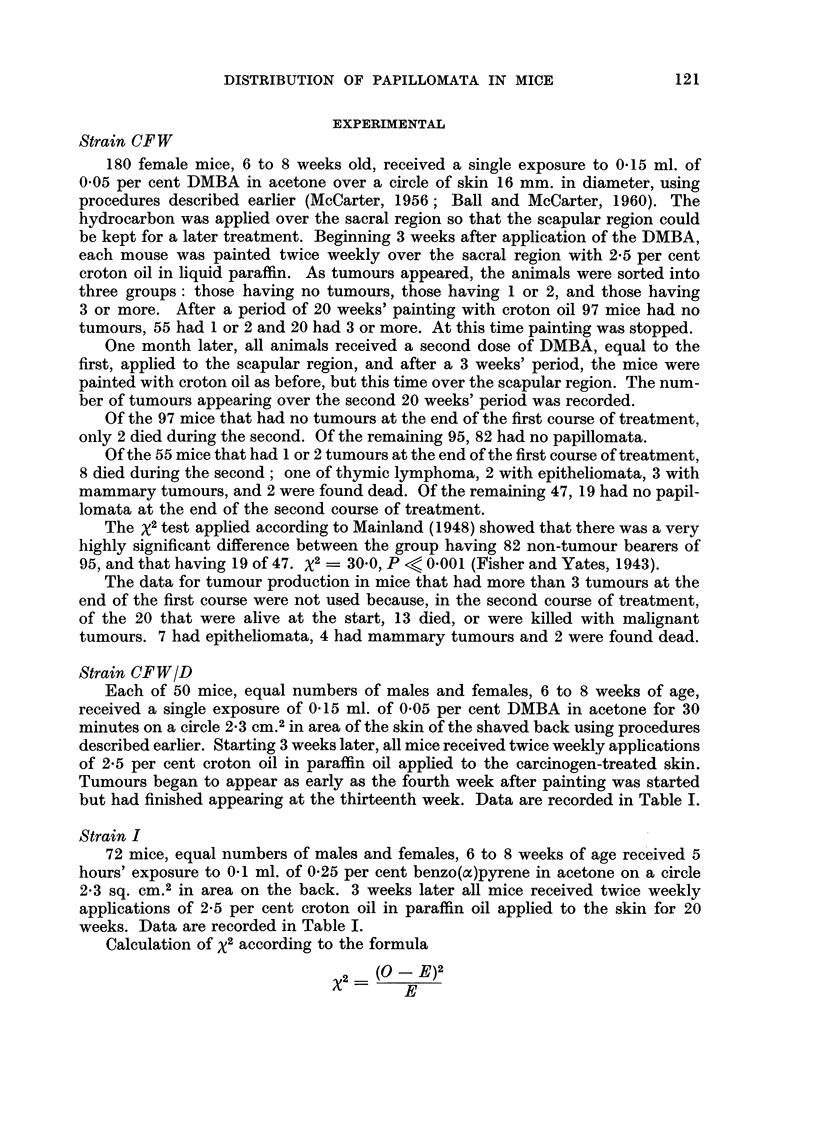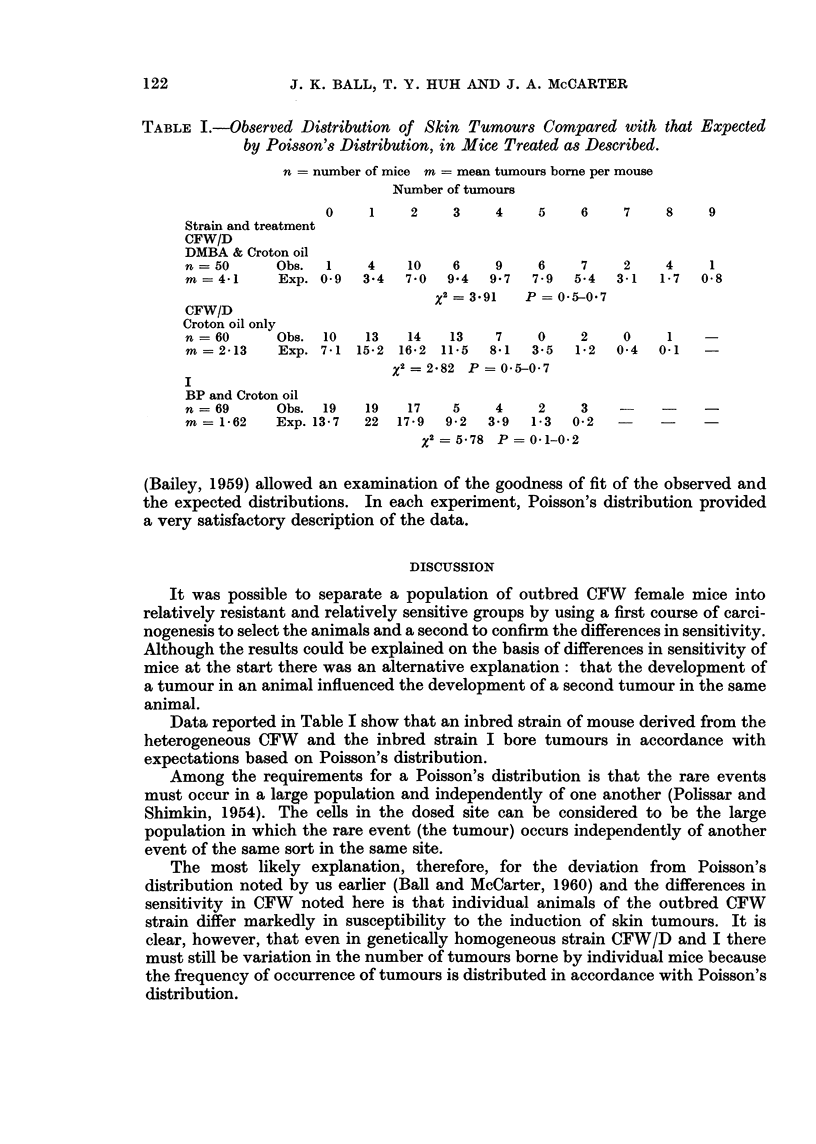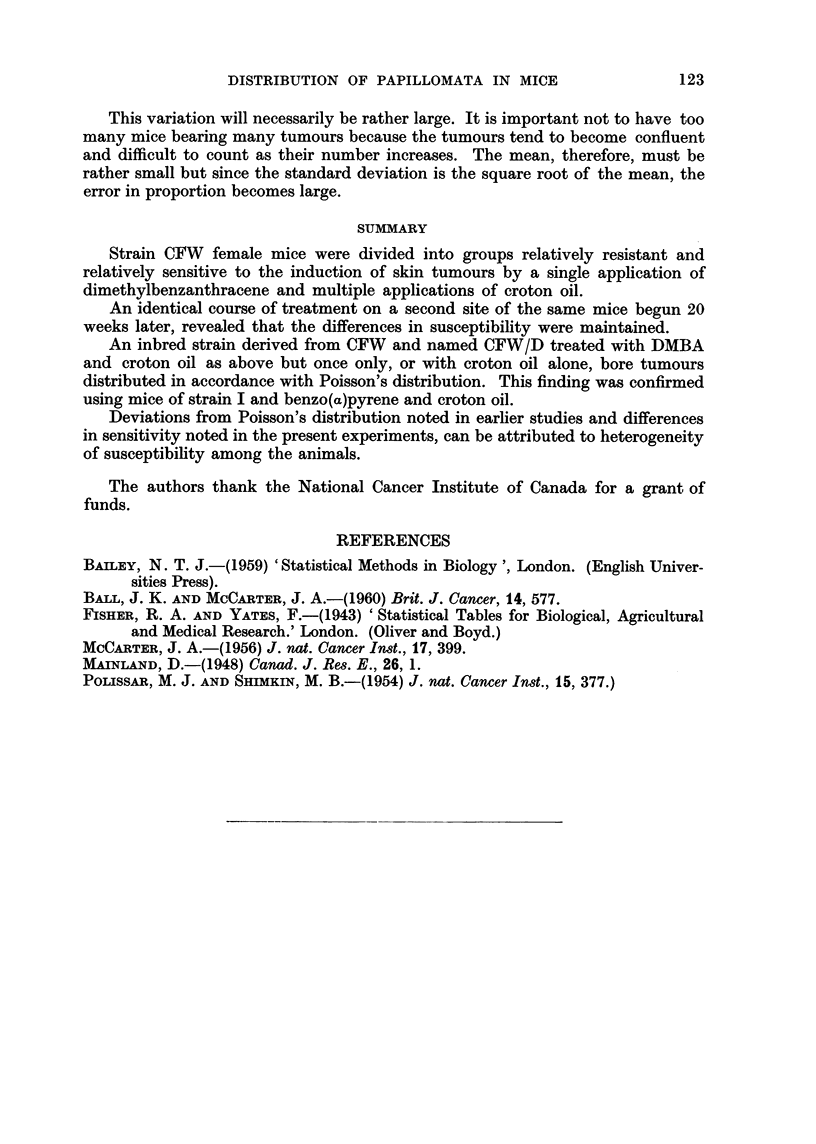# On the Statistical Distribution of Epidermal Papillomata in Mice

**DOI:** 10.1038/bjc.1964.12

**Published:** 1964-03

**Authors:** J. K. Ball, T. Y. Huh, J. A. McCarter


### ON THE STATISTICAL DISTRIBUTION OF EPIDERMAL PAPILLOMATA IN MICE

J. K. BALL, T. Y. HUH AND J. A. McCARTER

*From the Department of Biochemistry Dalhousie University, Halifax, Canada*

Received for publication January 27, 1964

IN a previous investigation reported from this laboratory (Ball and McCarter, 1960) it was noted that tumours produced in the skin of the CFW mouse by treatment with 7,12-dimethylbenz($\alpha$)anthracene (DMBA) and croton oil, were not distributed among the mice in accordance with the expected Poisson's distribution. Animals bearing no tumours and those bearing many were much more numerous than expected.

A quantitative analysis of induced primary adenomatous pulmonary tumours in mice was reported by Polissar and Shimkin (1954). They showed that the occurrence of such tumours was subject to Poisson's distribution and that deviations from this distribution could be attributed to heterogeneity of susceptibility in the animals.

We have now analyzed the data obtained in our laboratory in three populations of mice undergoing epidermal carcinogenesis.

### MATERIALS AND METHODS

*Strain CFW*.—These mice were females, purchased from Carworth Farms Inc., New City, New York. They were housed in groups of 10 in acrylic plastic boxes with stainless steel tops. The bedding was sawdust. Water and Purina Fox Chow Cubes were freely available.

*Strain CFW/D*.—This strain originated when, through error, a male was included among the female CFW mice purchased from the supplier in 1958. Since that time, brother-sister mating has been carried out with a view to obtaining a single inbred line. Litters selected for brother-sister mating were chosen on the basis of health, number in the litter and even distribution of the sexes and not for sensitivity to carcinogenesis. The mice were in the thirteenth and fourteenth inbred generations when used. They were cared for as described above.

*Strain I*.—This strain was obtained several years ago through the kindness of Dr. H. B. Andervont. The mice had been mated brother-to-sister for 71 to 72 generations when the experiment was begun.

*Chemicals*.—7,12-dimethylbenz($\alpha$)anthracene and benzo($\alpha$)pyrene were obtained from Eastman Organic Chemicals. Croton oil was obtained from Bush and Co., Canada. Paraffin oil viscosity 125/135 NF was a product of Fisher Scientific Co., Montreal, Canada. Meprobamate (Miltown) was kindly supplied by Dr. F. M. Berger, Wallace Laboratories, New Brunswick, N. J. Acetone, reagent grade, and diethyl ether, U.S.P. were distilled before use.

### EXPERIMENTAL

*Strain CFW*

180 female mice, 6 to 8 weeks old, received a single exposure to 0·15 ml. of 0·05 per cent DMBA in acetone over a circle of skin 16 mm. in diameter, using procedures described earlier (McCarter, 1956; Ball and McCarter, 1960). The hydrocarbon was applied over the sacral region so that the scapular region could be kept for a later treatment. Beginning 3 weeks after application of the DMBA, each mouse was painted twice weekly over the sacral region with 2·5 per cent croton oil in liquid paraffin. As tumours appeared, the animals were sorted into three groups: those having no tumours, those having 1 or 2, and those having 3 or more. After a period of 20 weeks' painting with croton oil 97 mice had no tumours, 55 had 1 or 2 and 20 had 3 or more. At this time painting was stopped.

One month later, all animals received a second dose of DMBA, equal to the first, applied to the scapular region, and after a 3 weeks' period, the mice were painted with croton oil as before, but this time over the scapular region. The number of tumours appearing over the second 20 weeks' period was recorded.

Of the 97 mice that had no tumours at the end of the first course of treatment, only 2 died during the second. Of the remaining 95, 82 had no papillomata.

Of the 55 mice that had 1 or 2 tumours at the end of the first course of treatment, 8 died during the second; one of thymic lymphoma, 2 with epitheliomata, 3 with mammary tumours, and 2 were found dead. Of the remaining 47, 19 had no papillomata at the end of the second course of treatment.

The $\chi^2$ test applied according to Mainland (1948) showed that there was a very highly significant difference between the group having 82 non-tumour bearers of 95, and that having 19 of 47. $\chi^2 = 30{\cdot}0$, $P \ll 0{\cdot}001$ (Fisher and Yates, 1943).

The data for tumour production in mice that had more than 3 tumours at the end of the first course were not used because, in the second course of treatment, of the 20 that were alive at the start, 13 died, or were killed with malignant tumours. 7 had epitheliomata, 4 had mammary tumours and 2 were found dead.

*Strain CFW/D*

Each of 50 mice, equal numbers of males and females, 6 to 8 weeks of age, received a single exposure of 0·15 ml. of 0·05 per cent DMBA in acetone for 30 minutes on a circle 2·3 cm.$^2$ in area of the skin of the shaved back using procedures described earlier. Starting 3 weeks later, all mice received twice weekly applications of 2·5 per cent croton oil in paraffin oil applied to the carcinogen-treated skin. Tumours began to appear as early as the fourth week after painting was started but had finished appearing at the thirteenth week. Data are recorded in Table I.

*Strain I*

72 mice, equal numbers of males and females, 6 to 8 weeks of age received 5 hours' exposure to 0·1 ml. of 0·25 per cent benzo($\alpha$)pyrene in acetone on a circle 2·3 sq. cm.$^2$ in area on the back. 3 weeks later all mice received twice weekly applications of 2·5 per cent croton oil in paraffin oil applied to the skin for 20 weeks. Data are recorded in Table I.

Calculation of $\chi^2$ according to the formula

$$\chi^2 = \frac{(O - E)^2}{E}$$

TABLE I.—*Observed Distribution of Skin Tumours Compared with that Expected by Poisson's Distribution, in Mice Treated as Described.*

$n$ = number of mice $m$ = mean tumours borne per mouse

| Strain and treatment | | Number of tumours 0 | 1 | 2 | 3 | 4 | 5 | 6 | 7 | 8 | 9 |
|---|---|---|---|---|---|---|---|---|---|---|---|
| CFW/D DMBA & Croton oil | | | | | | | | | | | |
| $n = 50$ | Obs. | 1 | 4 | 10 | 6 | 9 | 6 | 7 | 2 | 4 | 1 |
| $m = 4 \cdot 1$ | Exp. | 0·9 | 3·4 | 7·0 | 9·4 | 9·7 | 7·9 | 5·4 | 3·1 | 1·7 | 0·8 |
| | | | | $\chi^2 = 3 \cdot 91$ $P = 0 \cdot 5$–$0 \cdot 7$ | | | | | | | |
| CFW/D Croton oil only | | | | | | | | | | | |
| $n = 60$ | Obs. | 10 | 13 | 14 | 13 | 7 | 0 | 2 | 0 | 1 | — |
| $m = 2 \cdot 13$ | Exp. | 7·1 | 15·2 | 16·2 | 11·5 | 8·1 | 3·5 | 1·2 | 0·4 | 0·1 | — |
| | | | | $\chi^2 = 2 \cdot 82$ $P = 0 \cdot 5$–$0 \cdot 7$ | | | | | | | |
| I BP and Croton oil | | | | | | | | | | | |
| $n = 69$ | Obs. | 19 | 19 | 17 | 5 | 4 | 2 | 3 | — | — | — |
| $m = 1 \cdot 62$ | Exp. | 13·7 | 22 | 17·9 | 9·2 | 3·9 | 1·3 | 0·2 | — | — | — |
| | | | | $\chi^2 = 5 \cdot 78$ $P = 0 \cdot 1$–$0 \cdot 2$ | | | | | | | |

(Bailey, 1959) allowed an examination of the goodness of fit of the observed and the expected distributions. In each experiment, Poisson's distribution provided a very satisfactory description of the data.

### DISCUSSION

It was possible to separate a population of outbred CFW female mice into relatively resistant and relatively sensitive groups by using a first course of carcinogenesis to select the animals and a second to confirm the differences in sensitivity. Although the results could be explained on the basis of differences in sensitivity of mice at the start there was an alternative explanation : that the development of a tumour in an animal influenced the development of a second tumour in the same animal.

Data reported in Table I show that an inbred strain of mouse derived from the heterogeneous CFW and the inbred strain I bore tumours in accordance with expectations based on Poisson's distribution.

Among the requirements for a Poisson's distribution is that the rare events must occur in a large population and independently of one another (Polissar and Shimkin, 1954). The cells in the dosed site can be considered to be the large population in which the rare event (the tumour) occurs independently of another event of the same sort in the same site.

The most likely explanation, therefore, for the deviation from Poisson's distribution noted by us earlier (Ball and McCarter, 1960) and the differences in sensitivity in CFW noted here is that individual animals of the outbred CFW strain differ markedly in susceptibility to the induction of skin tumours. It is clear, however, that even in genetically homogeneous strain CFW/D and I there must still be variation in the number of tumours borne by individual mice because the frequency of occurrence of tumours is distributed in accordance with Poisson's distribution.

This variation will necessarily be rather large. It is important not to have too many mice bearing many tumours because the tumours tend to become confluent and difficult to count as their number increases. The mean, therefore, must be rather small but since the standard deviation is the square root of the mean, the error in proportion becomes large.

### SUMMARY

Strain CFW female mice were divided into groups relatively resistant and relatively sensitive to the induction of skin tumours by a single application of dimethylbenzanthracene and multiple applications of croton oil.

An identical course of treatment on a second site of the same mice begun 20 weeks later, revealed that the differences in susceptibility were maintained.

An inbred strain derived from CFW and named CFW/D treated with DMBA and croton oil as above but once only, or with croton oil alone, bore tumours distributed in accordance with Poisson's distribution. This finding was confirmed using mice of strain I and benzo(*a*)pyrene and croton oil.

Deviations from Poisson's distribution noted in earlier studies and differences in sensitivity noted in the present experiments, can be attributed to heterogeneity of susceptibility among the animals.

The authors thank the National Cancer Institute of Canada for a grant of funds.

### REFERENCES

Bailey, N. T. J.—(1959) 'Statistical Methods in Biology', London. (English Universities Press).

Ball, J. K. and McCarter, J. A.—(1960) *Brit. J. Cancer*, **14**, 577.

Fisher, R. A. and Yates, F.—(1943) 'Statistical Tables for Biological, Agricultural and Medical Research.' London. (Oliver and Boyd.)

McCarter, J. A.—(1956) *J. nat. Cancer Inst.*, **17**, 399.

Mainland, D.—(1948) *Canad. J. Res. E.*, **26**, 1.

Polissar, M. J. and Shimkin, M. B.—(1954) *J. nat. Cancer Inst.*, **15**, 377.)